# Cervical hibernoma in a two year old boy

**DOI:** 10.11604/pamj.2013.16.27.2063

**Published:** 2013-09-25

**Authors:** Khalid Khattala, Aziz Elmadi, Hanane Bouamama, Mohamed Rami, Youssef Bouabdallah

**Affiliations:** 1 Department of pediatric surgery, Hospital University Hassan II, Fez, Morroco

**Keywords:** Cervical, hibernoma, children, surgery

## Abstract

Hibernomas are uncommon benign soft tissue tumours mimicking brown fat. They are mostly seen in the fourth and fifth decades of life. Only few cases in the cervical area have been reported. Because of its rarity in pediatrics and diffcult diagnosis, we report a tow year-old patient with a cervical tumor. Ultrasound and computed tomography exams showed an infiltrative, with hypervascular and lipomatous features. After tumor excision, histopathological exam confirmed the diagnosis of hibernoma or brown fat tumor. This presentation describes the characteristics of this type of tumor, rare in children.

## Introduction

Hibernomas are uncommon benign soft tissue tumors, developed from fetal brown fat tissue, vestigial remnants of the evolution of the species. We present a case of neck hibernoma in a 2 year old boy patient and discuss the radiographic, pathologic, and clinical features of this tumor.

## Patient and observation

**Figure 1:**
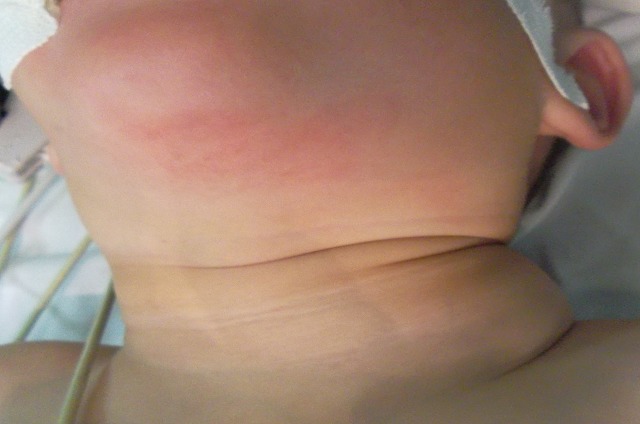
A supraclavicular basicervical tumour

**Figure 2:**
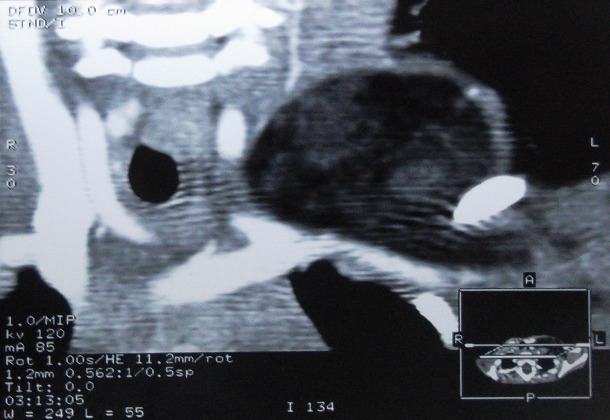
Cervical CT scann view of a heterogeneous, well-defined lesion with hypervascularity

**Figure 3:**
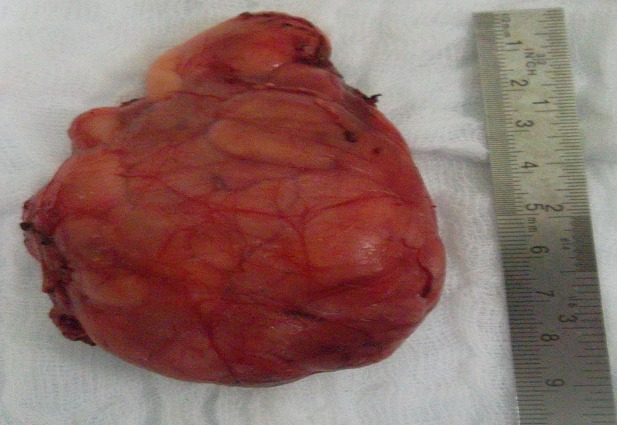
Macroscopic view of the specimen

**Figure 4:**
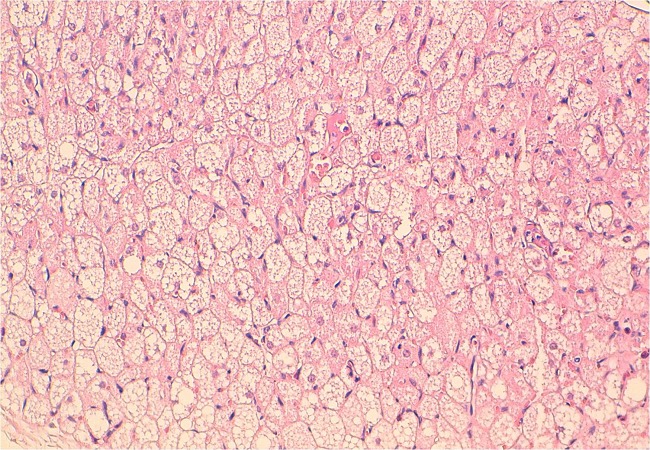
Histologic image: HESx250: multivacuolated brown fat cells with prominent nuclei

A two year old boy, presented with a six-month history of a left supraclavicular mass, which had increased in size over the previous twomonths prior to presentation. The swelling caused discomfort with movement but there were no neurological deficits, the patient did not have difficulty in swallowing or breathing, his medical history was unremarkable, Clinical examination revealed a mobile, soft mass compatible with a lipoma. This lesion measured approximately 9 cm in its maximum diameter; we found no cervical, supraclavicular or axillary node (Figure 1). Laboratory findings did not reveal anemia (haemoglobinemia = 13 g/dl) or inflammatory changes (sedimentation rate = 8mm, leucocytes 9600/mm3, C. Reactive Protein (CRP) = 0.8 mg/dl). On ultrasound, there was an ill-defined large heterogenous mass with increased vascularity. CT neck showed a 46 × 69 × 91 mm left supraclavicular mass posterior to the sternocleidomastoid muscle and superficial to the paravertebral muscles, of fatty density with fine internal septations (Figure 2 and Figure 3). The radiological appearance was suggestive of a lipoma. The mass was completely excised and examined histopathologically. Histopathologic investigation confirmed the diagnosis of benign hibernoma. Microscopically, numerous lobules separated by septa of connective tissue werepresent. Two tumoural cell types were seen: cells with granular deeply eosinophilic cytoplasm and clear multivacuolated cells filled with lipid droplets. The nuclei were always small, regular and round. There was no atypia and mitoses were rare (Figure 4), the tumour had a thick intact capsule consisting of collagenousconnective tissue. Postoperative recovery was uneventful and the patient was discharged on the thirth postoperative day, without any recurrence after one year of follow up.

## Discussion

Hibernoma is a rare benign soft tissue tumour composed of multiloculated fat cells derived from brown fat. This tumour was first described in 1906 by Merkl. The term hibernoma was proposed in 1914 by Gery because of its morphologic similarity to the cells of the so-called hibernating gland of animals [1, 2]

Brown adipose tissue, the function of which is to promote non-shivering thermogenesis. In man, it is found physiologically in newborns in the axillary and the subpleural regions but is said to disappear after 8 weeks of life [2]. In ourknowledge, only 12 cases of cervical location are reported [3], the cases reported in pubmed are listed in Table 1.

**Table 1:** Cervical hibernomas reported in PUBMED

Reference	Age	Sex	Size	Follow up
D Boudana and al [3]	26 years	male	7x8,6x5,6 cm	3 months
T Peycru and al[4]	43 years	male	12x8 cm	Two years
ErolBaskurt and al [6]	1month	female	3mm and 1,9x0,8 cm	
Minni A and al [7]	46 years	male	2cm	1 year

Hibernomas are mostly seen in the fourth and fifth decades of life, previously afemale predominancewas reported [1, 2, 4]. Clinically, their consistency is multiple but usually harder than a regular lipoma. It is a mobile, slow-growing mass. Thesymptoms are exceptional (pain or massive weight loss [3, 4].

Hibernomas are usually depicted as heterogeneous masses with marked contrast enhancement. The CT and MR imaging examinations show a well-demarcated mass with signal intensity intermediate between subcutaneous fat and muscle and that enhances after contrast injection. Although they present as brown fat, the imaging characteristics on T1- and T2-weighted images demonstrate high signal intensity but slightly less than that of the subcutaneous fat. On fat suppression sequences, there may be incomplete fat suppression because of the nature and amount of lipids. On MR imaging, flow voids can be identified, though not in this case. In T1inside the tumour, there were areas of intermediatesignals between that of the muscle and thesubcutaneous fat. Contrast enhancement was significantafter injection of gadolinium, particularlyin low-signal areas. In T2, the mass signal was oftenvery near that of the subcutaneous fat [1, 2, 5].

As needle biopsy carries a risk of haemorrhage and often leads to inconclusive results, definitive pathological diagnosisis based on surgical resection [1]. Upon microscopic examination, the tumors are characterized by cells of various degrees of differentiation. Multivacuolar adipocytes and brown fat cells with granular eosinophilic cytoplasm are interspersed with univacuolar adipocytes. Hypervascularity combined with abundant mitochondria give hibernomas their color. There are 4 histologic variants (typical, myxoid, lipomalike, and spindle cell), all of them with a benign course [4, 5].

Curative treatment of hibernomas is a complete excision, preserving vital structures, no case of recurrence has been reported [1, 2, 5].

## Conclusion

Hibernoma is a rare benign tumor, the cervical location is exceptionally, curative treatment is a complete excision, no recurrence has been reported.
